# Association of Persistent Symptoms after Lyme Neuroborreliosis and Increased Levels of Interferon-α in Blood

**DOI:** 10.3201/eid2906.221685

**Published:** 2023-06

**Authors:** Sergio A. Hernández, Katarina Ogrinc, Miša Korva, Andrej Kastrin, Petra Bogovič, Tereza Rojko, Keith W. Kelley, Janis J. Weis, Franc Strle, Klemen Strle

**Affiliations:** Tufts University School of Medicine, Boston, Massachusetts, USA (S.A. Hernández, K. Strle);; New York State Department of Health, Albany, New York, USA (S.A. Hernández, K. Strle);; University Medical Center Ljubljana, Ljubljana, Slovenia (K. Ogrinc, P. Bogovič, T. Rojko, F. Strle};; University of Ljubljana, Ljubljana (M. Korva, A. Kastrin);; University of Illinois, Urbana-Champaign, Illinois, USA (K.W. Kelley);; University of Utah, Salt Lake City, Utah, USA (J.J. Weis)

**Keywords:** Lyme neuroborreliosis, persistent symptoms, Lyme disease, immune response, inflammation, interferon-alpha, IFN-α, increased levels, post‒Lyme disease symptoms, tick-borne infections, vector-borne infections, blood, zoonoses, United States, Slovenia, *Suggested citation for this article*: Hernández SA, Ogrinc K, Korva M, Kastrin A, Bogovič P, Rojko T, et al. Association of persistent symptoms after Lyme neuroborreliosis and increased levels of interferon-α in blood. Emerg Infect Dis. 2023 Jun [*date cited*]. https://doi.org/10.3201/eid2906.221685

## Abstract

Patients who have Lyme neuroborreliosis (LNB) might experience lingering symptoms that persist despite antibiotic drug therapy. We tested whether those symptoms are caused by maladaptive immune responses by measuring 20 immune mediators in serum and cerebrospinal fluid (CSF) in 79 LNB patients followed for 1 year. At study entry, most mediators were highly concentrated in CSF, the site of the infection. Those responses resolved with antibiotic therapy, and associations between CSF cytokines and signs and symptoms of LNB were no longer observed. In contrast, subjective symptoms that persisted after use of antibiotics were associated with increased levels of serum interferon-α (IFN-α), which were already observed at study entry, and remained increased at each subsequent timepoint. Highest IFN-α levels corresponded with severe disease. Although the infection serves as the initial trigger, sequelae after antibiotic therapy are associated with unremitting systemic IFN-α levels, consistent with the pathogenic role of this cytokine in interferonopathies in other conditions.

Lyme disease, which is caused by several species of *Borrelia burgdorferi* sensu lato complex (Lyme borreliae), is the most common vectorborne disease in the Northern Hemisphere. The US Centers for Disease Control and Prevention now estimates that nearly 500,000 new cases are diagnosed and treated for Lyme disease annually in the United States (*1*). Approximately 200,000 cases are believed to occur each year in western Europe (*2*,*3*). The first sign of the infection is usually an expanding cutaneous lesion, termed erythema migrans, which might be accompanied with nonspecific symptoms, such as headache, fever, malaise, fatigue, myalgias, and arthralgias. If untreated, spirochetes might disseminate to other organ systems, including the heart, joints, and central nervous system (CNS) (*4*,*5*). Lyme neuroborreliosis (LNB), which results from borreliae infection of the CNS, is the most common extracutaneous manifestation of Lyme disease in Europe and the second most common such manifestation in North America (*4*–*6*).

Most patients who have Lyme disease respond well to antibiotic drug therapy and their disease resolves. However, a subset of patients have sequelae, such as headache, fatigue, sleep or memory disturbance, and arthralgias or myalgias that persist for months to years after antibiotic use, termed posttreatment Lyme disease symptoms or syndrome (PTLDS) (*7*–*14*). Those sequelae might occur with each Lyme disease manifestation and are heterogenous, ranging from mild, self-resolving symptoms to debilitating syndromes that greatly impair the quality of life and require regular use of analgesics (*7*–*13*,*15*). Although PTLDS is generally more common in women and in persons who have a difficult early disease course (*12*,*15*), the underlying causes are not well understood, and biomarkers to identify patients at risk for such outcomes are lacking. Thus, treatment strategies are symptom-based and often ineffective, leaving patients and physicians in a quandary about how to restore health.

Although persistent infection is often considered as an explanation for lingering symptoms, microbiological evidence of infection in the post‒antibiotic drug period is limited in humans. Two clinical trials in Europe of antibiotic drug efficacy established that only 2 (0.8%) of 244 patients had microbiologic treatment failure, and neither had PTLDS (*10*,*11*). Similarly, in xenodiagnostic studies, 2 of 11 patients with PTLDS were positive for borrelia DNA, but all were culture negative, implying lack of viable organisms (*16*). Findings from those microbiologic studies are substantiated by 5 clinical trials in United States and Europe that have failed to show sustained amelioration of PTLDS with additional antimicrobial drug therapy (*17*–*20*).

Emerging evidence now points to metabolic and immune system abnormalities in post-Lyme sequelae (*21*–*24*). Because Lyme borreliae do not encode toxins, the clinical manifestation of disease has been largely attributed to the host immune response. By extension, 2 longitudinal studies of erythema migrans patients demonstrated that sustained activation of this response could contribute to persistent symptoms in the post‒antibiotic drug period, when few, if any, spirochetes remain (*21*,*24*). A study of US patients by Aucott et al. reported increased serum levels of CCL19 in those with PTLDS, implying that ongoing immune reactivity in peripheral tissues might contribute to PTLDS (*21*). In our study of patients in Europe, a subset who had PTLDS had increased interleukin (IL) 23 levels in serum that correlated directly with autoantibodies but not with borrelia antibodies or culture positivity, thus implicating dysregulated Th17 immunity in those symptoms (*24*). Those reports linked the immune response to PTLDS after erythema migrans. However, the potential immune etiology of persistent symptoms after neurologic manifestations of Lyme disease has not been explored.

We investigated the role of host immune responses in the clinical course and outcome of LNB, a CNS infection with a high prevalence (≈20%) of post‒antibiotic drug sequelae. (*12*) We determined during infection whether immune responses in cerebrospinal fluid (CSF) correlated with signs and symptoms of LNB. In contrast, we also determined whether post-LNB sequelae were associated with increased interferon-α (IFN-α) levels in blood, supporting a role for unremitting, low-grade, systemic inflammation in those outcomes.

## Methods

### Patient Selection

This study included 79 adult patients who met the modified European diagnostic criteria for LNB (*4*,*14*), defined as presence of signs or symptoms indicative of neurologic involvement (i.e., radicular pain, cranial nerve paresis, meningeal signs/meningitis, tremor); CSF lymphocytic pleocytosis; and evidence of borrelial infection demonstrated by intrathecal synthesis of borrelia-specific antibodies, *Borrelia* isolation from CSF, or presence of erythema migrans. Patients were recruited on an ongoing basis in the Lyme borreliosis outpatient clinic at the University Medical Center Ljubljana, Ljubljana, Slovenia, during 2006‒2013. The 79 patients were chosen based on availability of matched serum and CSF samples for each patient at multiple follow-up timepoints. All participants provided written informed consent, and the study was approved by the Medical Ethics Committee of the Ministry of Health of Slovenia and the Institutional Review Boards at Massachusetts General Hospital and the New York State Department of Health.

### Clinical Assessment

We collected demographic and clinical information at baseline and at subsequent visits at 2 weeks and 3, 6, and 12 months after study entry by using a structured questionnaire (*12*). In addition to medical history, patients reported recurrence of tick bites, presence of EM, and severity and duration of symptoms. We also assessed signs of neurologic involvement (meningeal signs, cranial nerve damage, radiculitis, tremor) (*14*). In addition, we asked patients about any new or intensified (more severe) subjective symptoms.

We classified patients as resolved if they recovered completely with antibiotic therapy or as persistent symptoms/PTLDS if they had new or intensified subjective symptoms at 6 or 12 months after initiation of antibiotic therapy for which there was no other medical explanation. We further subgrouped patients who had PTLDS into 2 categories according to self-reported severity of symptoms based on a visual analog scale: those with mild-to-moderate symptoms that required occasional analgesics (once or twice weekly), and those who had debilitating symptoms with major impact on quality of life that required frequent use (>3×/wk) of analgesics. In addition to clinical assessment, we obtained serum at each time point and CSF at baseline and at 3 months. We obtained serum and CSF samples during the same study visit, enabling direct comparison. Samples were stored at −80°C. The same study physician evaluated all patients over the entire study period.

### Laboratory Evaluation

We assessed antibody levels (IgM and IgG) to *B. burgdorferi* sensu lato in serum and CSF by using an indirect chemiluminescence immunoassay against *OspC* and *VlsE* recombinant antigens (LIAISON; Diasorin, https://www.diasorin.com). We determined intrathecal borrelia IgM/IgG as described; antibody index values >1.5 were indicative of borrelia-specific antibody production (*25*). We defined CSF pleocytosis as CSF leukocyte counts >5 × 10^6^ cells/L and performed cultivation of borreliae from CSF as reported (*26*).

### Cytokine and Chemokine Determinations

We assessed levels of 20 cytokines and chemokines associated with innate (CCL2, CCL3, CCL4, IL-6, IL-8, IL-10, tumor necrosis factor-α, IFN-α) and adaptive T-cell (T_H_1: IFN-γ, CXCL9, CXCL10, CXCL11, CCL19, IL-12p70; T_H_17: IL-17A, IL-21, IL-23; CCL21) and B-cell (CXCL12, CXCL13) immune responses in serum and CSF by using bead-based multiplex assays (EMD-Millipore, https://www.emdmillipore.com). We performed cytokine determinations in all samples in 1 complete experiment to minimize interassay variation and repeated sample freeze‒thaw.

### Statistical Analyses

We assessed categorical variables by using the Fisher exact test and quantitative variables by using the Mann-Whitney nonparametric rank-sum test (GraphPad.PRISM 9.2.0, https://www.graphpad.com). We adjusted p values for multiple comparisons by using the Benjamini-Hochberg procedure. We used a robust, rank-based analysis of variance to examine differences in cytokine/chemokine levels in repeated measures and using time as a within-subject factor and severity as a between-subject factor; we considered p<0.05 statistically significant.

## Results

### Clinical Characteristics at Study Entry

A total of 79 patients were included in the study; 31 (39%) were female and 48 (61%) were male, and median age was 50 years (Table 1). Of the 79 LNB patients, 52 (66%) recovered completely after antibiotic therapy (resolved), whereas 27 (34%) had PTLDS 6–12 months after antibiotic therapy (persistent). The unusually high proportion of PTLDS patients is caused by our selection criteria, which we enriched for this patient population to enable meaningful comparisons. Compared with the resolved group, we found that patients who had PTLDS were more often female (59% vs. 41%; p = 0.03) and were more symptomatic at baseline (6 vs. 3 symptoms; p = 0.02) (Table 1). In contrast, we observed no major differences between these 2 groups for other demographic, laboratory, or clinical measurements, including the characteristic signs of LNB, Bannwarth syndrome or peripheral facial palsy.

**Table 1:** Clinical characteristics of patients at study entry stratified according to resolution or persistence of symptoms after antimicrobial drug therapy in study of persistent symptoms after Lyme neuroborreliosis and increased levels of interferon-α in blood, Slovenia*

Characteristic	Resolved, n = 52	Persistent, n = 27	p value†	Adjusted p value‡
Demographics				
Age, y	47 (15–81)	52 (15–74)	0.6	0.9
Female/male sex	15 (29)/37 (71)	16 (59)/11 (41)	0.01	**0.03**
Annual no. tick bites	2 (0–50)	1 (0–50)	0.6	0.9
Clinical characteristics at first visit				
Current or recent EM	19 (37)	9 (33)	0.8	0.9
Solitary EM	14 (27)	8 (30)	0.8	0.9
Multiple EM	5 (10)	1 (4)	0.6	0.9
No. symptoms/patient	3 (1–11)	6 (1–13)	0.02	0.1
Duration of symptoms, days	14 (3–90)	20 (3–365)	0.3	0.9
Radicular pain	14 (27)	9 (33)	0.6	0.9
Peripheral facial palsy	37 (71)	20 (74)	0.9	0.9
Laboratory findings in blood				
CRP, mg/L	1 (1–29)	1 (1–18)	0.2	0.6
ESR, mm/h	10 (2–40)	11 (1–41)	0.7	0.6
Blood leukocyte count, x 10^9^ cells/L	7 (3–23)	6.5 (3–15)	0.2	0.6
Platelet count, x10^9^/L	247 (134–428)	245 (165–416)	0.3	0.8
AST, µkat/L	.42 (0.18–1.1)	0.4 (0.24–1.2)	0.8	0.8
ALT, µkat/L	.43 (0.17–2.3)	0.39 (0.14–1.8)	0.5	0.8
CSF findings				
Pleocytosis	52 (100)	27 (100)	1	1
Leukocyte count, x 10^6^ cells/L	73 (6–1,579)	80 (6–806)	0.4	0.9
Lymphocyte count, x 10^6^/L	63 (4–1,477)	69 (4–768)	0.6	1
*Borrelia* culture positivity	5 (10)	3 (11)	0.9	0.9

*Values are median (range) or no. (%). Bold indicates statistically significant p values. ALT, alanine aminotransferase; AST, aspartate aminotransferase; *Borrelia, Borrelia burgdorferi* sensu lato; CRP, C-reactive protein; CSF, cerebrospinal fluid; EM, erythema migrans; ESR, erythrocyte sedimentation rate. †Statistical analyses were performed by using Fisher exact test or Mann-Whitney nonparametric rank-sum test. ‡Values after Benjamini-Hochberg correction for multiple comparisons (false discovery rate = 0.05).

### Prevalence of Constitutional Symptoms During Follow-up

**Figure 1:**
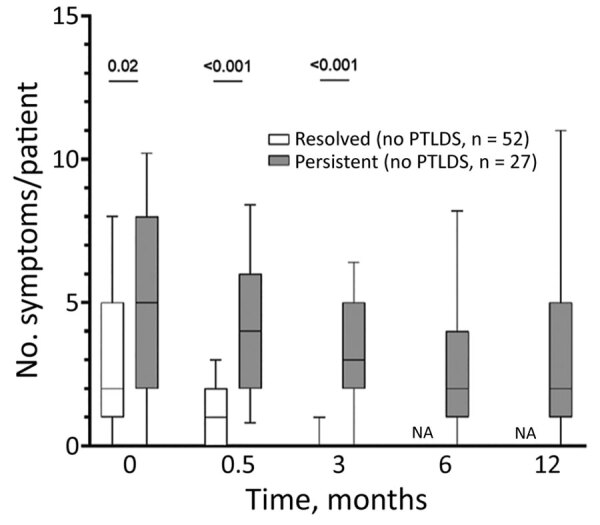
Individual frequency of each symptom at each follow-up timepoint in study of association of persistent symptoms after Lyme neuroborreliosis and increased levels of interferon-α in blood among patients in Slovenia. Data in patients whose symptoms resolved are shown in the left graph and in those with persistent symptoms in the right graph. Red stars indicate symptoms that occurred at significantly higher frequency in the persistent group compared with the resolved group. p values were calculated using nonparametric Mann-Whitney rank sum tests; p*<*0.05 was considered statistically significant. T, timepoint (0 indicates baseline).

**Figure 2:**
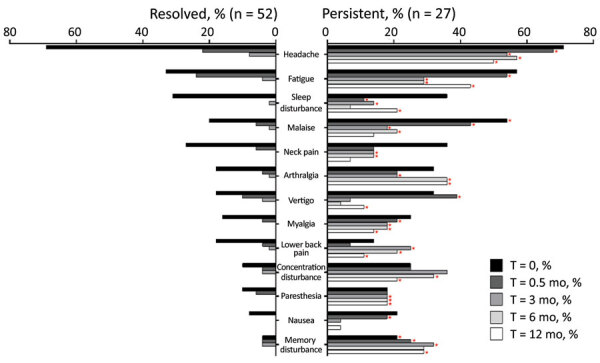
Prevalence of constitutional symptoms stratified according to posttreatment status in study of persistent symptoms after Lyme neuroborreliosis and increased levels of interferon-α in blood among patients in Slovenia.. Total number of symptoms in Lyme neuroborreliosis patients at each time point displayed according to resolved (n = 52, white box plots) or persistent (n = 27, gray box plots) symptoms [PTLDS]). Horizontal lines within boxes indicate medicans, box tops and bottoms 25th‒75th percentiles, and error bars 10th‒90th percentiles. Numbers above bars indicate p values. NA, not applicable; PTLDS, posttreatment Lyme disease symptoms or syndrome.

By definition, all LNB patients had signs and symptoms indicative of nervous system involvement (i.e., radiculitis, cranial nerve paresis, meningeal signs, tremor) at first visit, before antibiotic therapy. In addition to those neurologic signs and symptoms, patients often reported associated nonspecific systemic symptoms, most commonly headache, fatigue, and sleep disturbance (Figure 1). When stratified by presence of symptoms in the post‒antibiotic therapy period, patients who had PTLDS had a more symptomatic early disease course, before antibiotic therapy (6 vs. 3 symptoms; p = 0.02), as well as at the conclusion of therapy (after 2 weeks, 3.5 vs. 0 symptoms; p<0.001) and at 3 months (3 vs. 0 symptoms; p<0.001) (Figure 2). Moreover, when systemic symptoms were evaluated individually, 11 of the 13 symptoms were found at higher prevalence at the initial visit for patients who later had PTLDS develop, although only malaise and memory disturbance reached statistical significance (Figure 2). Those differences became more pronounced after antibiotic therapy; at 2 weeks, patients who had PTLDS had a higher prevalence of 9 of the 13 symptoms than did those in the resolved group (Figure 2).

### Comparison of Immune Mediators in Serum and CSF

We assessed levels of 20 cytokines and chemokines associated with innate and adaptive T- and B-cell immune responses in matched serum and CSF samples at study entry and 3 months. At baseline, 12 mediators were more highly concentrated in CSF, the site of infection, than in serum (Table 2). The highest levels and the most dramatic differences were observed for CCL2 (median 1,096 pg/mL vs. 668 pg/mL; p = 0.0002), the IFN-γ‒inducible chemokines CXCL9 (2,274 pg/mL vs 1,175 pg/mL; p = 0.05), and CXCL10 (16,920 pg/mL vs. 600 pg/mL; p = 0.0002), and B-cell chemoattractants CXCL12 (3,760 pg/mL vs. 2,875 pg/mL; p = 0.06) and CXCL13 (187 pg/mL vs. 18 pg/mL; p = 0.0002). Those data established that both innate and adaptive T- and B-cell immune responses are triggered during *Borrelia* infection and that those responses occur locally in CNS, the site of the disease. Conversely, TNF-α, CCL4, and IL-23 were present at greater levels in serum, suggesting that those responses occur systemically. However, by 3 months, most mediators decreased dramatically in CSF, reaching concentrations comparable to those in serum, presumably because of successful resolution of the CNS infection. Moreover, the concentrations of most mediators in serum were unremarkable and did not change between the 2 time points, further supporting the conclusion that immune responses occur locally in CSF and thereby contribute to CNS symptoms (Table 3).

**Table 2:** Comparison of cytokine and chemokine levels in cerebrospinal fluid and serum at study entry and 3 mo for association of persistent symptoms after Lyme neuroborreliosis and increased levels of interferon-α in blood, Slovenia *

Mediator	T = 0, n = 719				T = 3 mo, n = 64		
	Median CSF (range), pg/mL	Median serum (range), pg/mL	p value†		Median CSF (range), pg/mL	Median serum (range), pg/mL	p value†
Innate							
IFN-α	3 (0–64)	2 (0–187)	1		1 (0–19)	2 (0–158)	**0.0001**
IL-6	7 (0–146)	1 (0–234)	**0.0002**		1 (0–1)	1 (0–88)	0.1
IL-10	6 (0–422)	1 (0–40)	**0.0002**		1 (0–1)	1 (0–3)	1
IL-8	144 (18–1,288)	23 (0–2,159)	**0.0002**		31 (13–107)	15 (1–1,700)	**0.0002**
TNF	10 (0–150)	20 (2–385)	**0.0002**		2 (0–20)	19 (2–278)	**0.0001**
CCL2	1,096 (223–48,494)	668 (60–1,579)	**0.0002**		719 (241–1,889)	688 (287–7,311)	0.3
CCL3	49 (1–107)	25 (1–1,310)	**0.003**		38 (1–87)	21 (1–1080)	**0.0001**
CCL4	29 (1–787)	127 (29–2,567)	**0.0002**		11 (1–35)	129 (33–1,223)	**0.0001**
T_H_1 adaptive							
IFN-γ	2 (0–193)	3 (0–349)	1		1 (0–11)	1 (0–32)	**0.0001**
CXCL9	2,274 (14–37,051)	1,175 (49–26,310)	**0.05**		117 (1–1,997)	853 (110–20,472)	**0.0001**
CXCL10	16,920 (137–139,804)	600 (66–4,484)	**0.0002**		905 (201–16,407)	439 (50–6,221)	**0.0001**
CXCL11	80 (1–2,640)	45 (1–534)	**0.003**		10 (1–86)	46 (1–499)	**0.0001**
CCL19	55 (10–1,598)	54 (10–939)	0.5		10 (3–362)	73 (7–1,730)	**0.0001**
IL-12p70	3 (1–17)	3 (1–1,486)	0.5		1 (1–3)	3 (1–45)	**0.0001**
T_H_17 adaptive							
IL-17a	9 (4–25)	4 (0–347)	**0.0002**		7 (3–11)	5 (1–64)	**0.0001**
IL-21	3 (3–11)	3 (3–229)	0.5		20 (1–20)	20 (3–41)	0.1
IL-23	3 (3–408)	13 (0–13,296)	**0.007**		49 (0–85)	49 (0–2,123)	**0.02**
CCL21	530 (312–927)	97 (20–1,473)	**0.0002**		530 (256–1,222)	98 (20–1,323)	**0.0001**
B-cell adaptive							
CXCL12	3,760 (98–27,479)	2,875 (98–16,039)	**0.006**		1,953 (98–6,880)	2,875 (98–16,039)	**0.02**
CXCL13	187 (1–107,723)	18 (5–172)	**0.0002**		4 (1–62)	13 (1–151)	**0.0001**

*Bold indicates statistically significant p values. CCL, CC motif chemokine ligand; CSF, cerebrospinal fluid; CXCL, CXC motif chemokine ligand; IFN, interferon; IL, interleukin; T, timepoint (0 indicates baseline); TNF, tumor necrosis factor. †Benjamini-Hochberg corrected (false discovery rate = 0.05).

**Table 3:** Association of inflammatory mediators in cerebrospinal fluid and serum at study entry with posttreatment Lyme disease symptoms 6 or 12 mo after acute illness for association of persistent symptoms after Lyme neuroborreliosis and increased levels of interferon-α in blood, Slovenia*

Mediator	Cerebrospinal fluid, n = 67				Serum, n = 78		
	Median (range) PTLDS, pg/mL, n = 24	Median (range) no PTLDS, pg/mL, n = 43	p value†		Median (range) PTLDS, pg/mL, n = 27	Median (range) no PTLDS, pg/mL, n = 51	p value†
Innate							
IFN-α	10 (0–64)	3 (0–64)	0.8		18 (0–187)	2 (0–129)	**0.05**
IL-6	3 (1–73)	8 (0–146)	0.9		1 (1–234)	1 (0–115)	1
IL-10	127 (22–1,288)	168 (18–860)	0.9		32 (1–381)	20 (0–2,159)	0.5
IL-8	4 (0–241)	6 (0–422)	0.8		1 (0–7)	1 (0–40)	0.5
TNF	9 (0–98)	10 (0–150)	0.9		17 (7–164)	20 (2–385)	0.4
CCL2	985 (223–48,494)	1,111 (311–6,099)	0.9		697 (245–1,234)	650 (60–1,579)	0.6
CCL3	45 (1–95)	50 (1–107)	0.8		24 (1–268)	26 (1–1,310)	0.8
CCL4	29 (1–787)	29 (1–105)	1		154 (65–1,405)	116 (29–2,567)	0.4
T_H_1 adaptive							
IFN-γ	3 (0–193)	2 (0–30)	0.9		3 (0–349)	1 (0–24)	0.3
CXCL9	1,524 (29–21,800)	2,370 (14–37,051)	0.9		916 (433–3,819)	1,396 (49–26,310)	0.7
CXCL10	10,410 (260–118,875)	17,794 (137–139,804)	0.9		555 (231–4,484)	622 (66–3,422)	0.8
CXCL11	74 (2–2,645)	86 (1–1,194)	0.9		10 (1–151)	66 (2–534)	**0.05**
CCL19	68 (10–1,062)	53 (10–1,598)	0.9		31 (10–484)	74 (10–939)	**0.05**
IL-12p70	1 (1–17)	3 (1–12)	0.9		3 (1–1,486)	1 (1–21)	0.2
T_H_17 adaptive							
IL-17a	10 (5–25)	8 (4–21)	0.8		6 (1–347)	4 (1–13)	0.1
IL-21	3 (3–10)	3 (3–11)	0.9		3 (3–10)	3 (3–229)	**0.05**
IL-23	3 (3–47)	3 (3–498)	0.9		22 (0–931)	9 (0–13,296)	0.8
CCL21	563 (376–804)	530 (312–927)	0.9		98 (20–989)	95 (20–1,473)	0.8
B-cell adaptive							
CXCL12	4,384 (98–27,479)	2,950 (98–24,080)	0.9		2,882 (98–9,061)	2,868 (98–16,039)	0.8
CXCL13	272 (5–107,723)	114 (5–50,000)	0.8		24 (5–123)	18 (5–172)	0.6

*Bold indicates statistically significant p values. CCL, CC motif chemokine ligand; CSF, cerebrospinal fluid; CXCL, CXC motif chemokine ligand; IFN, interferon; IL, interleukin; PTLDS, posttreatment Lyme disease symptoms; TNF, tumor necrosis factor. †Benjamini-Hochberg corrected (false discovery rate = 0.05).

### Immune Responses at the Initial Visit According to PTLDS

Because the greater frequency of symptoms at first visit was associated with an unfavorable clinical outcome, we assessed whether the immune responses during early infection might predict resolution or persistence of symptoms after antibiotic drug therapy. For that purpose, we stratified cytokine and chemokine levels at initial visit, before use of antimicrobial drugs, by resolution or persistence of symptoms 6–12 months later (Table 4). In serum, 4 mediators varied greatly between patients who had persistent or resolved symptoms. Levels of 3 mediators, CXCL11, CCL19, and IL-21, were higher in patients whose symptoms resolved after antibiotic therapy, implying that they might play a protective role in the infection and contribute to symptom resolution. Those associations were lost in follow-up (data not shown), substantiating their role in the infection. In contrast, IFN-α levels were significantly higher in patients who had PTLDS (median 18 pg/mL vs. 2 pg/mL; p = 0.01). A similar trend was observed for IL-17A and IL-23, but those differences were not significant after adjustment for multiple comparisons. In CSF, none of the mediators differentiated patients with persistent symptoms from those whose symptoms resolved, despite the association between increased CSF immune responses, symptoms, and CNS infection. This finding implies the decoupling of immune responses in CSF during infection from systemic immune responses associated with the development of PTLDS in the post‒antibiotic therapy period.

**Table 4:** Cytokine and chemokine levels at study entry vs 3 mo in cerebrospinal fluid and serum for association of persistent symptoms after Lyme neuroborreliosis and increased levels of interferon-α in blood, Slovenia*

Mediator	Cerebrospinal fluid				Serum		
	T = 0, median (range), pg/mL, n = 67	T = 3, median (range), pg/mL, n = 64	p value†		T = 0, median (range), pg/mL, n = 78	T = 3, median (range), pg/mL, n = 74	p value†
Innate							
IFN-α	3 (0–64)	1 (0–19)	**0.0005**		2 (0–187)	2 (0–158)	0.7
IL-6	7 (0–146)	1 (0–1)	0.6		1 (0–234)	1 (0–88)	**0.0007**
IL-8	144 (18–1,288)	1 (0–1)	**0.001**		1 (0–40)	15 (1–1,700)	**0.0007**
IL-10	6 (0–422)	31 (13–107)	0.4		23 (0–2,159)	1 (0–3)	0.2
TNF	10 (0–150)	2 (0–20)	**0.001**		20 (2–385)	19 (2–278)	0.8
CCL2	1,096 (223–48,494)	719 (241–1,889)	**0.001**		668 (60–1,579)	688 (287–7,311)	0.4
CCL3	49 (1–107)	38 (1–87)	**0.001**		25 (1–1,310)	21 (1–1,080)	0.8
CCL4	29 (1–787)	11 (1–35)	**0.001**		127 (29–2,567)	129 (33–1,223)	0.8
T_H_1 adaptive							
IFN-γ	2 (0–193)	1 (0–11)	**0.003**		3 (0–349)	1 (0–32)	0.8
CXCL9	2,274 (14–37,051)	117 (1–1,997)	**0.001**		1,175 (49–26,310)	853 (110–20,472)	**0.03**
CXCL10	16,920 (137–139,804)	905 (201–16,407)	**0.001**		600 (66–4,484)	439 (50–6,221)	**0.03**
CXCL11	80 (1–2,640)	10 (1–86)	**0.001**		45 (1–534)	46 (1–499)	0.9
CCL19	55 (10–1,598)	10 (3–362)	**0.001**		54 (10–939)	73 (7–1,730)	0.7
IL-12p70	3 (1–17)	1 (1–3)	**0.001**		3 (1–1,486)	3 (1–45)	0.2
T_H_17 adaptive							
IL-17a	9 (4–25)	7 (3–11)	**0.001**		4 (0–347)	5 (1–64)	0.9
IL-21	3 (3–11)	20 (1–20)	**0.009**		3 (3–229)	20 (3–41)	**0.0007**
IL-23	3 (3–408)	49 (0–85)	**0.003**		13 (0–13,296)	49 (0–2,123)	**0.005**
CCL21	530 (312–927)	530 (256–1,222)	**0.001**		97 (20–1,473)	98 (20–1,323)	0.9
B-cell adaptive							
CXCL12	3,760 (98–27,479)	1,953 (98–6,880)	0.2		2,875 (98–16,039)	2,875 (98–16,039)	0.8
CXCL13	187 (1–107,723)	4 (1–62)	**0.001**		18 (5–172)	13 (1–151)	0.2

*Bold indicates statistically significant p values. CCL, CC motif chemokine ligand; CSF, cerebrospinal fluid; CXCL, CXC motif chemokine ligand; IFN, interferon; IL, interleukin; T, timepoint (0 indicates baseline); TNF, tumor necrosis factor. †Benjamini-Hochberg corrected (false discovery rate = 0.05).

### Immune Responses During Follow-up According to PTLDS

**Figure 3:**
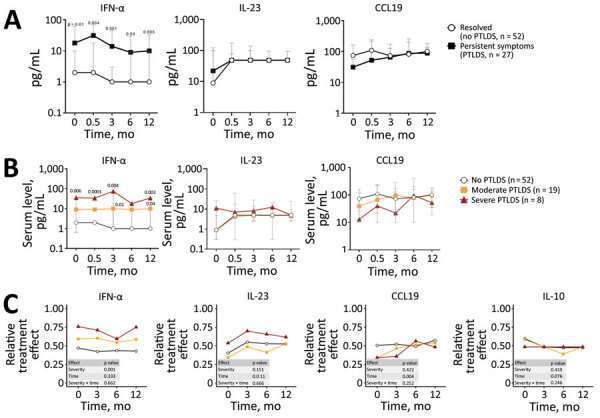
Cytokine and chemokine levels in serum of patients in Slovenia who had Lyme neuroborreliosis in study of association of persistent symptoms after Lyme neuroborreliosis and increased levels of interferon-α in blood. Prevalence of constitutional symptoms is stratified according to PTLDS severity during the 1-year follow-up period. A) Median serum levels of IFN-α, IL-23, and CCL19 during 1 year of follow-up stratified by presence or absence of PTLDS. B) Median serum levels of IFN-α, IL-23, and CCL19 graphed according to disease severity. p values above red line correspond to the comparison between patients with severe PTLDS vs those with no PTLDS (resolved). p values above yellow lines represent significant differences between the no PTLDS and moderate PTLDS groups. C) Analysis of variance comparison of relative effect of disease severity or time on cytokine levels in serum. Error bars indicate 10^th^–90^th^ percentiles CIs. CCL, CC motif chemokine ligand; IFN, interferon; IL, interleukin; PTLDS, posttreatment Lyme disease symptoms or syndrome.

IFN-α in serum was the only mediator that remained significantly increased in patients with PTLDS at each follow-up visit, compared with patients whose symptoms resolved (Figure 3, panel A). Similar results were observed when participants were further stratified according to PTLDS severity (Figure 3, panel B). Patients with severe symptoms had the highest levels of IFN-α in serum at baseline (median 35.5 pg/mL), those with moderate severity had intermediate IFN-α concentrations (9 pg/mL), and those without PTLDS had the lowest IFN-α levels (2 pg/mL) in serum. This association was observed at each subsequent time point. A similar trend was observed for IL-23, although those differences did not reach statistical significance. In contrast, other mediators did not differ by disease severity at any time point after antibiotic therapy, including CCL19, which was previously associated with PTLDS in patients after erythema migrans (*21*), and IL-10, which is a major regulatory cytokine (Figure 3, panel C). A nonparametric analysis of variance-type testing, which measured the effect of relative disease severity on cytokine levels, reinforced this association between PTLDS, IFN-α, and IL-17/IL-23 pathways (Figure 3, panel C).

**Figure 4:**
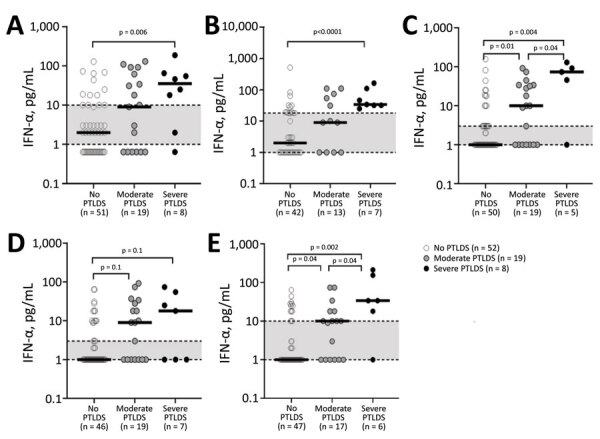
Serum IFN-α levels of individual patients with Lyme neuroborreliosis in Slovenia at each follow-up timepoint according to PTLDS severity in study of association of persistent symptoms after Lyme neuroborreliosis and increased levels of interferon-α in blood. A) T = 0; B) T = 2 wks; C) T = 3 mo; D) T = 6 mo; E) T = 12 mo. Levels of interferon-α in serum for individual patients throughout the 1-year follow-up are shown. Solid black lines symbolize median values, and shaded area between dotted lines indicates interquartile range. Statistical analyses were performed by using nonparametric Mann-Whitney rank-sum tests. Significant p values for each comparison are shown above the corresponding brackets. IFN, interferon; PTLDS, posttreatment Lyme disease symptoms or syndrome.

We provide serum IFN-α levels for individual patients (Figure 4). Although there was overlap between disease severity groups, most patients with severe PTLDS had IFN-α levels above the third quartile of patients without PTLDS at each time point. Thus, increased serum IFN-α levels in the post‒antibiotic therapy period might play a pathogenic role by contributing to ongoing symptoms after LNB.

## Discussion

A subset of patients with LNB experience lingering symptoms that persist despite appropriate antimicrobial therapy. The etiology of such symptoms is unclear, and effective biomarkers and treatment strategies are lacking. The usefulness of this report is that it defines IFN-α as a distinguishing marker and possibly a mediator of post-Lyme sequelae. These findings have potential implications for diagnosis of patients and treatment for this condition.

Prolonged systemic symptoms have been observed after several bacterial and viral infections, including infectious mononucleosis, influenza, Q fever, and COVID-19 (*27*,*28*). Despite the diversity of triggering agents, several common themes have emerged. As a generalization, those sequelae comprise a constellation of nonspecific symptoms that occur more frequently after severe early infection; they also are believed to represent a postinfectious process and are indistinguishable by standard clinical, demographic, or laboratory markers (*27*). Those observations are also applicable to patients who have Lyme disease, including LNB, ≈10%–20% of whom report lingering symptoms after antimicrobial drug therapy for *Borrelia* infection (*7*–*13*). Combined, those studies suggest that, despite different triggers, the underlying pathophysiology of postinfectious sequelae might be similar.

In the absence of microbiological evidence of persistent infection, other hypotheses have been proposed to explain lingering symptoms after Lyme disease. Those hypotheses include retained spirochetal antigens (*29*,*30*), metabolic irregularities (*31*), and excessive inflammation (*21*,*23*,*24*), all of which could arguably be linked to inappropriate activation of host immune responses. Insights from our study of patients with LNB, along with 2 previous reports of patients with erythema migrans who were followed longitudinally to evaluate their posttreatment status (*21*,*24*), support the role of maladaptive immune responses in PTLDS. Moreover, those studies suggest that the stage for dysregulated immunity is set early in infection. In all 3 studies, heightened levels of PTLDS-associated inflammatory mediators were observed at first visit, before antimicrobial drug therapy, implying that *Borrelia* infection serves as the initial trigger for these responses (*21*,*24*). However, the sustained immune activity in the post‒antibiotic therapy period, which occurs concurrently with post-Lyme sequelae, appears to represent a postinfectious process (*10*,*11*,*24*). Similar observations have been made in patients with postinfectious Lyme arthritis who have persistent synovitis for months to years after receiving antibiotics (*23*,*32*). Thus, a range of post-Lyme complications have been linked to dysregulated host immunity.

What is driving unremitting inflammation in the absence of an ongoing infection is not yet entirely clear. One possibility is that spirochetal remnants, such as *Borrelia* peptidoglycan, which is not easily cleared, continue to stimulate immune responses in the postinfectious period (*29*,*30*). However, given that such components presumably also persist in patients whose symptoms resolve after treatment with antibiotics, this finding alone is unlikely the entire explanation for post-Lyme sequelae. Alternatively, several studies in Lyme arthritis point to infection-induced autoimmune phenomena as a potential reason for persistent postinfectious arthritis (*23*). Finally, in mice and in humans, genetic predisposition might contribute to dysregulated immunity (*33*–*35*). Those possibilities, and probably other unidentified mechanisms, are not mutually exclusive. Depending on the condition, multiple factors might play a role. This concept is supported at least partially by distinct immune signatures associated with PTLDS across different patient populations, including CCL19 in erythema migrans patients in the United States (*21*), IL-23 in erythema migrans patients in Europe (*24*), and IFN-α in patients who have LNB. Nevertheless, the preponderance of evidence across distinct manifestations underscores the role of maladaptive immune responses as both effectors and biomarkers of post‒antibiotic therapy sequelae after Lyme disease.

In this longitudinal study, both CSF and serum were available at multiple time points over 1 year. This approach enabled tracking of immunologic responses systemically and locally in CSF during the clinical course and outcome of LNB. This analysis showed that, during CNS infection, the innate and adaptive immune responses are highly concentrated in CSF, the site of the disease. Those responses resolve after antimicrobial drug therapy and presumed resolution of CNS infection. This contraction of immune responses in CSF coincides with resolution of neurologic signs and symptoms of LNB (radiculitis, cranial nerve damage, meningeal signs/meningism, tremor), thereby linking the infection and inflammation in CSF with CNS disease. In contrast, the nonspecific lingering symptoms after LNB are associated with increased IFN-α levels in serum. Those results imply that sustained systemic immune responses and associated symptomology are decoupled from infection-driven immune responses in CSF. This interpretation is consistent with the notion that type 1 IFNs represent a maladaptive immune response that contributes to disease pathology but not to control of *Borrelia* infection. In murine models, type 1 IFN responses are associated with development of arthritis in the absence of spirochetal eradication (*36*,*37*). In humans, increased IFN-α activity has been observed in patients with a history of Lyme disease and persistent cognitive deficits months to years after receiving antimicrobial drugs (*17*). Those observations provide new considerations for treatment approaches that prioritize targeting the immune response after appropriate antibiotic regimens, a treatment algorithm used successfully in post‒antibiotic therapy for Lyme arthritis (*23*,*38*).

Although this study does not establish causality, the association between increased systemic IFN-α levels and PTLDS is nonetheless intriguing. This cytokine plays a central role in maladaptive hyperinflammatory immune responses in type 1 interferonopathies in other infections and autoimmune diseases, including COVID-19, influenza, and lupus (*39*). The pathologic effects of IFN-α are also apparent from treatment studies in cancer and chronic viral hepatitis in which administration of this cytokine often results in adverse events, particularly influenza-like symptoms, such as fever, chills, myalgia, headache, and nausea, as well as neurologic and psychiatric sequelae (*40*,*41*). Those symptoms, which are remarkably similar to those in patients with PTLDS, occur in most patients undergoing IFN-α therapy and might lead to discontinuation of treatment. Lessons learned from these studies could offer major insights into novel therapeutic approaches that target IFN-α in Lyme disease. However, before such strategies can be considered, our initial observations need to be validated in larger cohorts of patients with PTLDS and appropriate control groups. Nevertheless, emerging anti‒IFN-α therapies offer hope for more effective treatment strategies for patients with debilitating lingering symptoms after LNB and perhaps other manifestations of Lyme disease.
